# Nucleus Accumbens-Associated Protein 1 Expression Has Potential as a Marker for Distinguishing Oral Epithelial Dysplasia and Squamous Cell Carcinoma

**DOI:** 10.1371/journal.pone.0131752

**Published:** 2015-07-14

**Authors:** Joji Sekine, Eiji Nakatani, Koichiro Ohira, Katsumi Hideshima, Takahiro Kanno, Yoshiki Nariai, Tatsuo Kagimura, Takeshi Urano

**Affiliations:** 1 Department of Oral and Maxillofacial Surgery, Shimane University Faculty of Medicine, Izumo, Shimane, Japan; 2 Translational Research Informatics Center, Foundation for Biomedical Research and Innovation, Kobe, Hyogo, Japan; 3 Department of Biochemistry, Shimane University Faculty of Medicine, Izumo, Shimane, Japan; Queen Mary Hospital, HONG KONG

## Abstract

**Background:**

Oral epithelial dysplasia (OED) and carcinoma in situ (CIS) are defined by dysplastic cells in the epithelium. Over a third of oral squamous cell carcinoma (OSCC) patients present with associated OED. However, accurate histopathological diagnosis of such lesions is difficult. Nucleus accumbens-associated protein 1 (NAC1) is a member of the Pox virus and Zinc finger/Bric-a-brac Tramtrack Broad complex family of proteins, and is overexpressed in OSCC. This study aimed to determine whether NAC1 has the potential to be used as a marker to distinguish OED and OSCC.

**Methods and Findings:**

The study included 114 patients (64 men, 50 women). There were 67, 10, and 37 patients with OED, CIS, and OSCC, respectively. NAC1 labeling indices (LIs) and immunoreactivity intensities (IRI) were evaluated. The patients’ pathological classification was significantly associated with age, sex, NAC1 LIs, and NAC1 IRI (p = 0.025, p = 0.022, p < 0.001, and p < 0.001, respectively). As a result of multivariate analysis, a predictive model was made; this identified the NAC1 LIs (OR [95% CI] 1.18 [1.11–1.28], p < 0.001) and NAC1 IRI (0.78 [0.68–0.86], p < 0.001) as predictive factors for CIS/OSCC. The NAC1 LIs/IRI cut-off values which discriminated between OED and CIS/OSCC were 50%/124 pixels. For NAC1 LIs with > 50% positivity the sensitivity, specificity, positive predictive value (PPV), and negative predictive value (NPV) were 0.766, 0.910, 0.857, and 0.847, respectively. For NAC1 IRI with ≤ 124 positive pixels, the sensitivity, specificity, PPV, and NPV were 0.787, 0.866, 0.804, and 0.853, respectively. Though there are several potential limitations to this study and the results were obtained from a retrospective analysis of a single site cohort, the data suggest that the NAC1 LIs/IRI is a strong predictor of CIS/OSCC.

**Conclusions:**

NAC1 has potential as a marker for distinguishing OED from CIS/OSCC.

## Introduction

Oral squamous cell carcinoma (OSCC) is commonly preceded by a range of tissue and cellular alterations that are consistent with carcinoma but are restricted to the surface epithelial layer; this is termed oral epithelial dysplasia (OED). Various attempts have been made to uniformly diagnose and discretely categorize the continuous range of tissue changes observed in OED and OSCC. Many of these have been based on the classification of precursor lesions in other epithelial sites, including squamous intraepithelial neoplasia (SIN) of the cervix [1].

However, the suitability of such classification systems for OED is limited. The most recently accepted classification developed by the World Health Organization divides OED into mild, moderate, severe, and carcinoma in situ (CIS) [2] based on histopathological assessment of various architectural and cytological deviations. CIS is defined by dysplastic epithelial cells extending from the basal layer to the mucosal surface, with features of malignancy [3]. However, an accurate diagnosis of such lesions is difficult in the clinical field [4], and even with histopathological specimens, the differential diagnosis of OED from intraepithelial lesions is problematic [3].

Although histopathology is recognized as the gold standard in the diagnosis of various oral lesions, the use of histopathology for the diagnosis and categorization of OED is considered imprecise [1]. Furthermore, more than a third of OSCC patients present with OED in close proximity. Various biological markers for malignant transformation and OED progression have been reported [5–10]. However, for maxillofacial surgeons, OED and SIN are both candidate lesions for resection, and given the aforementioned background, a simple and feasible marker for distinguishing OED from malignancy is required for application in routine work.

Nucleus accumbens-associated protein 1 (NAC1) is a member of the Pox virus and Zinc finger/Bric-a-brac Tramtrack Broad complex family of proteins that mediates several cellular functions, including proliferation, apoptosis, transcriptional control, and the maintenance of cell morphology [11]. NAC1 has been reported to be overexpressed in several types of human carcinoma [12], and we have also reported that NAC1 is overexpressed in OSCC cells from various different oral lesions [13]. Therefore, we hypothesized that NAC1 might be a useful marker to distinguish OED and OSCC. The aim of this study, which is a subgroup analysis of existing data from a previous report [13], was to determine whether NAC1 expression has potential as a marker for the differential diagnosis of OED, CIS, and OSCC.

## Materials and Methods

### Patients

The Shimane University Institutional Committee on Ethics approved the whole study (Approval No. 995; March 26, 2012, No. 996; March 28, 2012). This study is not necessary for approval of the authors' Institutional Review Board, because it have using a hospital database of Shimane University that has been unlinked anonymous. The unlinked anonymity of the patients was ensured by the president of Shimane University faculty of medicine.

Subjects underwent biopsy, with written and/or verbal informed consent, at the Department of Oral and Maxillofacial Surgery, Shimane University Hospital. Patients were histopathologically diagnosed with OED, CIS, or OSCC between 2007 and 2014. All histopathological data were retrospectively collected from the Department of Oral and Maxillofacial Surgery, Shimane University Faculty of Medicine.

### Tissue samples and NAC1 immunohistochemistry

Biopsy specimens taken from the margin of the lesion were fixed with 10% neutral buffered formalin for 24 h prior to routine processing. Paraffin-embedded sections were stained with hematoxylin and eosin and histopathologically diagnosed by pathology specialists in the Department of Pathology, Shimane University Hospital.

For immunohistochemical staining, sections were deparaffinized, rehydrated, and incubated in 0.3% hydrogen peroxide in methanol for 30 min to quench endogenous peroxidase activity. Antigen retrieval was performed by autoclaving in phosphate buffered salts (pH 7.4, TAKARA BIO Inc., Shiga, Japan); 10 phosphate buffered salt tablets were dissolved in distilled water and made up to a final volume of 1,000 mL (9.57 mM, pH 7.35–7.65). All immunohistochemistry sections were incubated with 10% rabbit serum blocking agent to block nonspecific reactions prior to primary antibody treatment.

After incubation with the NAC1 mouse monoclonal antibody (diluted 1:1,000, overnight at 4°C [11]), sections were incubated with the streptavidin/biotin reagent from the HISTOFINE SAB-PO (M) KIT (Nichirei, Tokyo, Japan) prior to application of a 0.05% diaminobenzidine tetrahydrochloride substrate solution. Counterstaining was performed with Mayer’s hematoxylin for 30 s. Immunohistochemistry negative control sections were incubated with phosphate buffered saline instead of primary antibody; no positive reactions were observed. Ovarian carcinoma sections, provided by Nakayama et al. [12], were used as a positive control. A positive reaction was confirmed following incubation with the NAC1 mouse monoclonal antibody (diluted 1:1,000, overnight at 4°C) [13]; the positive control staining is shown in S1 Fig.

### NAC1 labeling indices and NAC1 immunoreactivity intensity

All sections were examined by the third author using a standard light microscope with a × 40 objective lens. An attached digital camera was used to capture images and estimate the number of NAC1-positive cells. In OED and CIS, the average NAC1 labeling indices (LIs; labeled cells/total cells counted × 100%) was determined by examining the bottom-most 20 basal cells (the cells from the basal to the keratinized layer in the field of view) in at least 10 sites for each subject. In OSCC patients with no structure to the basal cell layers, at least 100 cells, including NAC1-positive and NAC1-negative cells, were counted at the invasive front of the lesion, in accordance with our previous study [13].

The nuclear margin of NAC1-positive cells (at least 100 cells) was delineated under high-magnification view (× 40 objective lens) using a standard light microscope to ensure accuracy. The NAC1 immunoreactivity intensity (IRI) was evaluated by analyzing the brightness of each pixel in RGB images using ImageJ v1.47 (National Institute of Health, Bethesda, MD); high values indicate a weak immunoreactivity and low values indicate a strong immunoreactivity.

### Statistical analysis

Continuous variables, such as age, NAC1 LIs, and NAC1 IRI, were stratified according to a pathological classification with six levels: mild OED, moderate OED, severe OED, CIS, well-differentiated OSCC, and moderately/poorly differentiated OSCC. For continuous variables, comparisons were made using the one-way analysis of variance F-test, and for categorical variables the Chi-squared test was used. To produce a new pathological classification consisting of two groups which benefit from the NAC1 LIs and IRI, the cut-off values for the six-level pathologic classification system were determined using the maximum t-test p-value. As a result of this, the six-level pathological classification was reclassified into two categories (OED and CIS/OSCC). To further explore any predictive factors associated with the two-level pathological classification, univariate and multivariate logistic regression analyses were performed. The odds ratio (OR) and 95% confidence interval (CI), based on the Wald test, were calculated. The multivariate analysis was performed using a backward selection method. To determine the cut-off for the NAC1 LIs and IRI, the maximum p-value from the t-tests for the two-level pathological classification was used. To evaluate cut-offs for the NAC1 index and intensity, we calculated the predictive performance measures (sensitivity, specificity, positive predictive value [PPV], and negative predictive value [NPV]). A p-value < 0.05 was considered significant. All statistical analyses were performed using SAS version 9.3 (Cary, NC, USA).

## Results

There were 114 patients included (50 men and 64 women) in the study and their ages ranged from 34 to 91 years (mean, 67.5 years). The number of OED, CIS, and OSCC patients was 67, 10, and 37, respectively (S1, S2 and S3 Tables). The lesion sites were the tongue (62 patients), gingiva (36 patients), buccal mucosa (11 patients), palate (2 patients), lip (2 patients), and the oral floor (1 patient). The severity of OED was classified as mild (34 patients), moderate (20 patients), or severe (13 patients). The TNM classification and pathological N staging of the OSCC patients is shown in S3 Table.

### NAC1 expression

**Fig 1:**
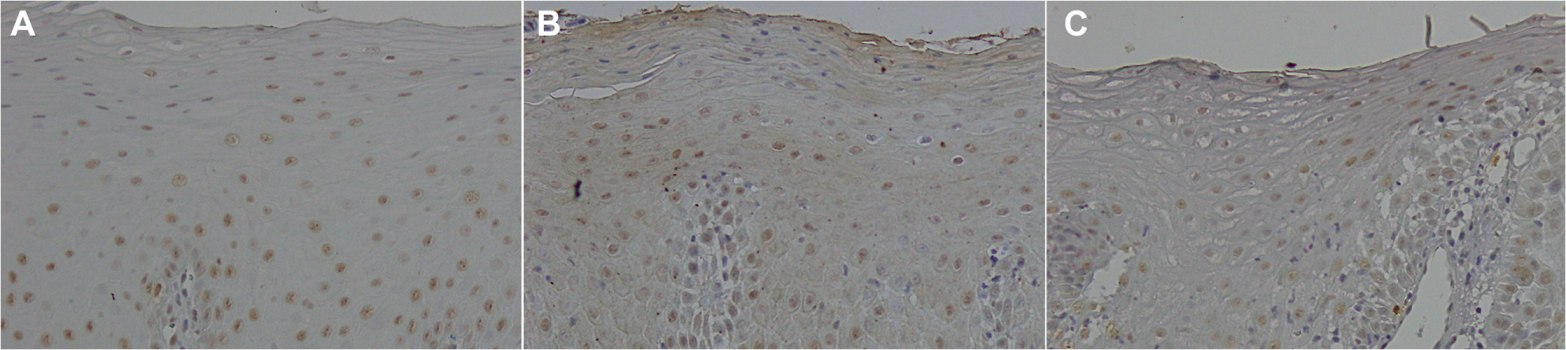
Nucleus accumbens-associated protein 1 (NAC1) expression.

In OED and CIS, NAC1-positive cells were predominantly distributed in the basal cell to spinous cell layers. However, NAC1-positive cells were also found in the proliferating area of OSCC (Fig 1 and S2 Fig). The NAC1 LIs were 37.8 ± 14.7%, 24.7 ± 15.0%, and 26.7 ± 16.6% in mild, moderate, and severe OED, respectively. The NAC1 LIs were 58.1 ± 15.2% in CIS and 57.3 ± 12.1% and 58.57 ± 13.2% in well- and moderately/poorly-differentiated OSCC. The NAC1 IRI was 134.8 ± 7.1, 128.2 ± 10.9, and 133.4 ± 9.1 pixels in mild, moderate, and severe OED, respectively. In CIS, the NAC1 IRI was 120.8 ± 6.8 pixels. In OSCC, the NAC1 IRI was 118.2 ± 7.0 and 120.9 ± 7.7 pixels in well- and moderately/poorly-differentiated cases, respectively.

In oral epithelial dysplasia (OED) and carcinoma in situ (CIS), NAC1-positive cells were distributed mainly from the basal cell to spinous cell layer. NAC1-positive cells were also found in the proliferating area of oral squamous cell carcinomas (OSCC).

A: OED (× 20). B: CIS (× 20). C: OSCC (× 20). Images of immunohistochemical staining for each OED, CIS, and OSCC are shown in S2 Fig.

### Distribution of NAC1 expression and its association with pathological classification

Table 1 shows the association between the pathological classification and patient characteristics and NAC1 expression. The pathological classification was significantly associated with patient age, sex, NAC1 LIs, and NAC1 IRI (p = 0.025, p = 0.022, p < 0.001, and p < 0.001, respectively; Table 1). When the data was reclassified from the six-level pathological classification into the simple two-category classification, t-tests indicated a further significant difference between the NAC1 LIs and IRI of OED and CIS/OSCC (p < 0.001).

**Table 1:** Association between pathological classification and patient characteristics and NAC1 expression.

		Classification		Univariate logistic regression			
Variable	Category or Unit	OED (N = 67)	OSCC/CIS (N = 47)	Odds Ratio	96% Cl	p-value	p-value of overall test
Age	1	68.4-/+12.0	66.1-/+12.8	0.98	0.95–1.02	0.325	0.325
Sex	Woman	38	12	1			0.001
	Men	29	35	3.82	1.73–8.89	0.001	
Tumor site	Tongue	37	25	1			0.356
	Gingiva	18	18	1.48	0.65–3.41	0.353	
	Buccal	8	3	0.56	0.11–2.13	0.417	
	Others	4	1	0.37	0.02–2.69	0.386	
NAC1 LI	1	31.7-/+16.2	57.8-/+12.9	1.14	1.09–1.20	<0.001	<0.001
NAC1 IRI	<50 or50	61	11	1			<0.001
	>50	6	36	33.27	12.10–106.41	<0.001	
NAC1 IRI	1	132.5-/+9.1	119.5-/+7.2	0.84	0.78–0.89	<0.001	<0.001
NAC1 IRI	<124 or 124	9	37	1			<0.001
	>124	58	10	0.04	0.01–0.11	<0.001	

Table 1 shows the mean +/− standard deviation for continuous variables and the number of patients for categorical variables. “Other” tumor sites include the lip, palate, and mouth floor. P-values were calculated using the F-test for continuous variables and Chi-squared test for categorical variables. NAC1: nucleus accumbens-associated protein 1, LI: labeling index, IRI: immunoreactive intensity, OED: oral epithelial dysplasia, CIS: carcinoma in situ, OSCC: oral squamous cell carcinoma, diff.: differentiated.

### Discrimination of OED and CIS/OSCC by NAC1 expression

In univariate logistic regression analysis, OED and CIS/OSCC were significantly associated with sex, NAC1 LIs, and NAC1 IRI. The ORs (95% CI; p-value) for sex, NAC1 LIs, and NAC1 IRI were 3.82 (1.73–8.89; p = 0.001), 1.14 (1.09–1.20; p < 0.001), and 0.84 (0.78–0.89; p < 0.001), respectively (Table 2). Of note, the results of a subset analysis stratified by sex showed similar results (S4 Table). Furthermore, as a result of multivariate analysis, a predictive model was produced: the model identified NAC1 LIs (OR [95% CI] 1.18 [1.11–1.28], p < 0.001) and NAC1 IRI (0.78 [0.68–0.86], p < 0.001) as predictive factors associated with CIS/OSCC (Table 3).

**Table 2:** Univariate regression analysis of OED and CIS/OSCC classification.

		Classification		Univariate logistic regression			
Variable	Category or Unit	OED (N = 67)	OSCC/CIS (N = 47)	Odds Ratio	96% Cl	p-value	p-value of overall test
Age	1	68.4-/+12.0	66.1-/+12.8	0.98	0.95–1.02	0.325	0.325
Sex	Woman	38	12	1			0.001
	Men	29	35	3.82	1.73–8.89	0.001	
Tumor site	Tongue	37	25	1			0.356
	Gingiva	18	18	1.48	0.65–3.41	0.353	
	Buccal	8	3	0.56	0.11–2.13	0.417	
	Others	4	1	0.37	0.02–2.69	0.386	
NAC1 LI	1	31.7-/+16.2	57.8-/+12.9	1.14	1.09–1.20	<0.001	<0.001
NAC1 IRI	<50 or50	61	11	1			<0.001
	>50	6	36	33.27	12.10–106.41	<0.001	
NAC1 IRI	1	132.5-/+9.1	119.5-/+7.2	0.84	0.78–0.89	<0.001	<0.001
NAC1 IRI	<124 or 124	9	37	1			<0.001
	>124	58	10	0.04	0.01–0.11	<0.001	

NAC1: nucleus accumbens-associated protein 1, LI: labeling index, IRI: immunoreactive activity, OED: oral epithelial dysplasia, CIS: carcinoma in situ, OSCC: oral squamous cell carcinoma, CI: confidence interval.

**Table 3:** Multivariate regression analysis of OED and CIS/OSCC classification.

Variable	Unit	Multivariate logistic regression		
		Odds Ratio	96% Cl	p-value
NAC1 LI	1	1.18	1.11–1.28	<0.001
NAC1 IRI	1	0.78	0.68–0.86	<0.001

NAC1: nucleus accumbens-associated protein 1, OED: oral epithelial dysplasia, CIS: carcinoma in situ, OSCC: oral squamous cell carcinoma, LI: labeling index, IRI: immunoreactive intensity, CI: confidence interval.

**Fig 2:**
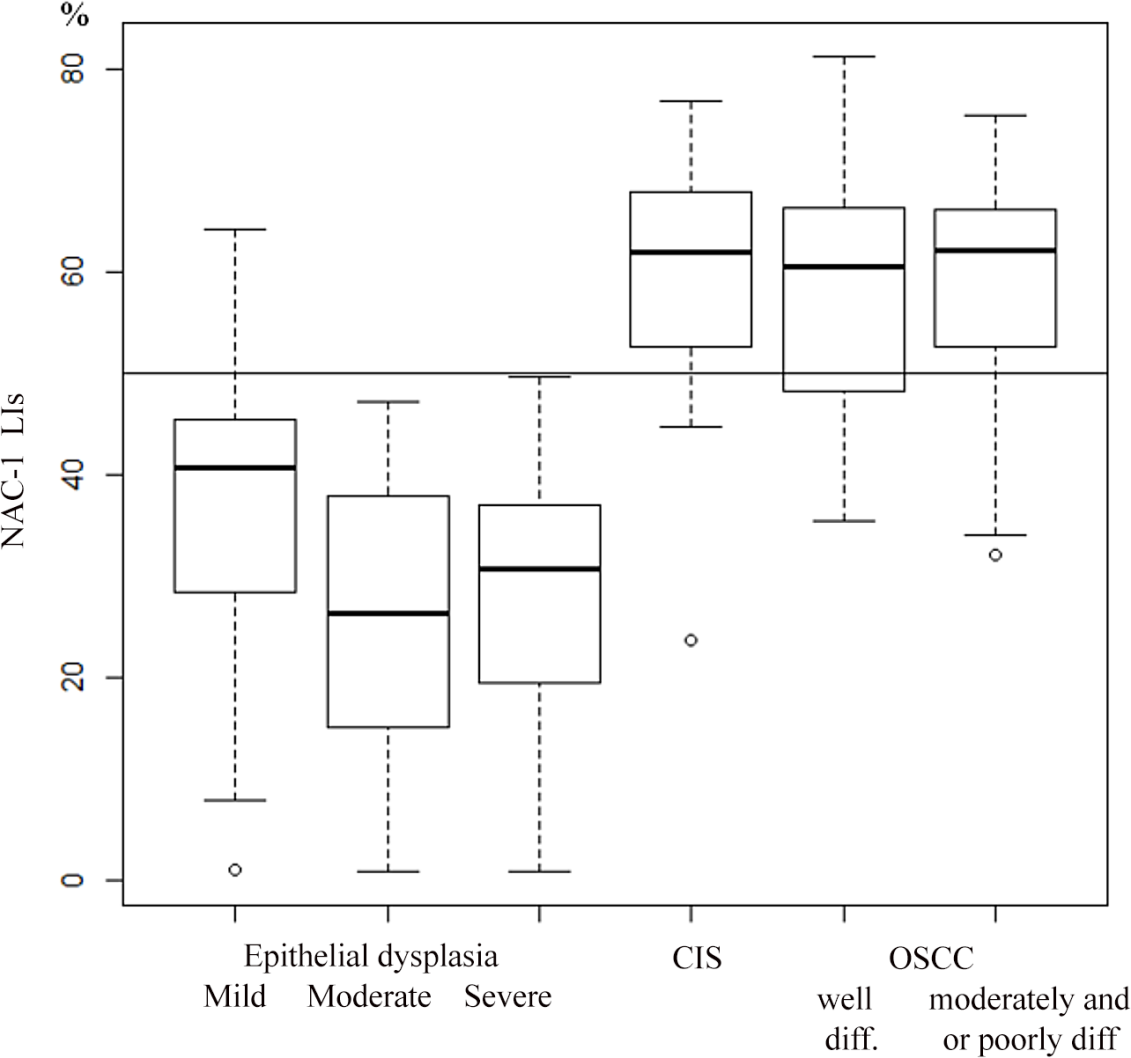
NAC1 labeling index of OED and CIS/OSCC samples.

**Fig 3:**
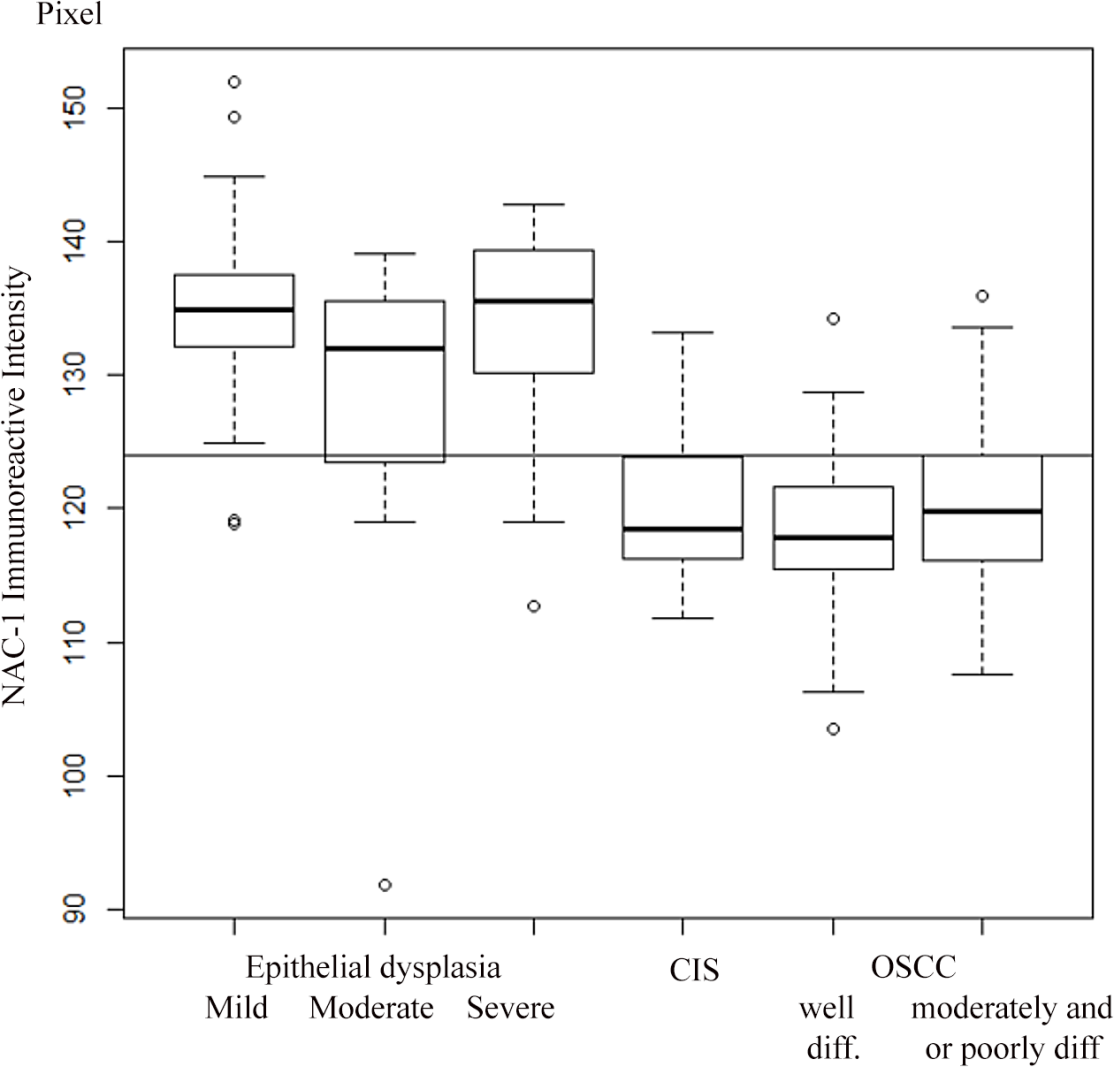
NAC1 immunoreactivity intensity of OED and CIS/OSCC samples.

The NAC1 LIs and NAC1 IRI cut-off values which discriminated between OED and CIS/OSCC were 50% and 124 pixels, respectively (Figs 2 and 3, and S5 and S6 Tables). For NAC1 LIs with > 50% positivity, the sensitivity, specificity, PPV, and NPV for CIS/OSCC were 0.766, 0.910, 0.857, and 0.847, respectively. For NAC1 IRI with ≤ 124 positive pixels, the sensitivity, specificity, PPV, and NPV were 0.787, 0.866, 0.804, and 0.853, respectively.

## Discussion

Histologically, OED can be divided into different levels of severity. However, the severity of OED is not always associated with risk of malignant transformation; the current histopathological OED grading system is not useful for predicting OCSS transformation [1]. OSCC develops through a multistep process whereby genetic mutations related to cell proliferation, differentiation, and tumor suppression accumulate. During this process, cancer precursor lesions can be morphometrically recognized [7]. Various different biomarker studies have been reported to date [5–10]. Nakabayashi et al. [5] have suggested that paired-like homeodomain 1 (PITX1) suppression is associated with OED malignant transformation. Using one of the largest multicenter OED cohorts, 4 biomarkers (EGFR, CD151, CD9, and COX2) have also been reported to have prognostic value for determining the malignant transformation of OED [6]. Moreover, the feasibility of using markers such as cyclin D1, p27, and p53 as predictors of OED malignant transformation have also been investigated [7]. Recently, a correlation between high human papilloma virus expression and oral malignancy was also reported [9,10]. However, some of these markers require sample analysis using PCR, real time-PCR, or *in situ* hybridization [8–10], but simple techniques using routine formalin-fixed paraffin-embedded biopsy specimens are required. Therefore, we have focused on NAC1 immunohistochemistry to evaluate its potential as a marker for distinguishing between OED and CIS/OSCC.

In this study, sex was shown to be a statistically significant determinant of CIS/OSCC (Table 1). Worldwide, head and neck squamous cell carcinoma is the sixth most malignant tumor, and it is estimated that there are 650,000 new cases every year [14]. A general trend of a high incidence rates in males (> 10/100,000) has been reported [15]. In multivariate regression analyses, sex was not identified as a significant factor, indicating that it is a poor and indirect predictor of CIS/OSCC (Table 2). However, in our study, multivariate regression analysis did identify NAC1 LIs and IRI as variables which can be used to distinguish OED and CIS/OSCC (Table 3).

Generally, dysplastic changes in the oral intraepithelial cells are associated with transformation from normal to malignant tissue [16]. The more dysplastic the epithelium becomes, the more the atypical epithelial changes tend to involve the entire thickness of the epithelium. Van Zyl et al. [17] showed that aneuploidy peaks in patients of mild dysplasia were predominantly in a peri-diploid position, and that the mean DNA index values gradually increased in line with the severity of the OED. The histopathological alterations characteristic of dysplastic epithelial cells are often similar to those of OSCC [3], making histopathological diagnosis difficult and potentially inaccurate.

NAC1 is considered to have oncogenic potential [18], and NAC1 expression is thought to be upregulated during the process of carcinogenesis. NAC1 has recently been identified as an important transcriptional regulator which functions as part of an extended regulatory network to maintain the pluripotency of various cells [19,20]. As such, it is logical that NAC1 can be used to aid the histological determination of OED and CIS/OSCC.

There are several potential limitations to the present study; in particular, this was a retrospective study using a cohort from a single site. However, our data from 114 patients collected over 8 years are sufficient to indicate that NAC1 LIs and IRI are strong predictors of CIS/OSCC. Future validation studies are needed to confirm the NAC1 cut-off values, but our results indicate that the NAC1 LIs and IRI are feasible means of categorizing OED to CIS/OSCC into two groups.

## Conclusions

NAC1 has the potential to be used as a biomarker for distinguishing OED from CIS/OSCC. Future validation studies are required to confirm the specific NAC1 cut-off values required.

## Supporting Information

S1 Fig Images of NAC1 immunohistochemical staining in ovarian carcinoma negative and positive controls. A: Negative control (× 20). B: Positive control (× 20).
(TIF)

S2 Fig Images of NAC1 immunohistochemical staining for each OED, CIS, and OSCC. A: Mild OED (× 20). B: Moderate OED (× 20). C: Severe OED (× 20). D: CIS (× 20). E: Well-differentiated OSCC (× 20). F: Moderately-differentiated OSCC (× 20). G: Poorly-differentiated OSCC (× 20).
(TIF)

S1 Table Detailed information on OED patients. (XLSX)

S2 Table Detailed information on CIS patients. (XLSX)

S3 Table Detailed information on OSCC patients. (XLSX)

S4 Table Association between the pathological classification and NAC1 expression, stratified by sex. (XLSX)

S5 Table P-values from t-tests for each cut-off value of the NAC1 LIs. (XLSX)

S6 Table P-values from t-tests for each cut-off value of the NAC1 IRI. (XLSX)
