# Supplementary figures and images for: Nucleus Accumbens-Associated Protein 1 Expression Has Potential as a Marker for Distinguishing Oral Epithelial Dysplasia and Squamous Cell Carcinoma
Source: PLoS One. 2015 Jul 14;10(7):e0131752. doi: 10.1371/journal.pone.0131752 (PMC4501714; doi:10.1371/journal.pone.0131752)

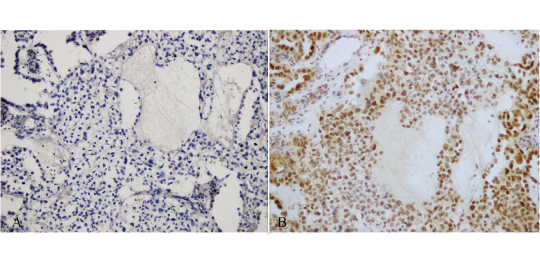

Supplement: S1 Fig — A: Negative control (× 20). B: Positive control (× 20). (TIF) [file pone.0131752.s001.tif]

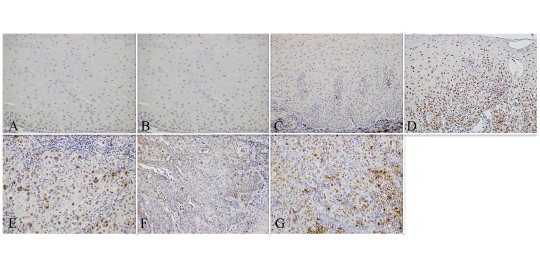

Supplement: S2 Fig — A: Mild OED (× 20). B: Moderate OED (× 20). C: Severe OED (× 20). D: CIS (× 20). E: Well-differentiated OSCC (× 20). F: Moderately-differentiated OSCC (× 20). G: Poorly-differentiated OSCC (× 20). (TIF) [file pone.0131752.s002.tif]
